# Improved Part-of-Speech Prediction in Suffix Analysis

**DOI:** 10.1371/journal.pone.0076042

**Published:** 2013-10-04

**Authors:** Mario Fruzangohar, Trent A. Kroeger, David L. Adelson

**Affiliations:** 1 School of Molecular & Biomedical Science, University of Adelaide, Adelaide, South Australia, Australia; 2 School of Computer Science, University of Adelaide, Adelaide, South Australia, Australia; Université de Nantes, France

## Abstract

**Motivation:**

Predicting the part of speech (POS) tag of an unknown word in a sentence is a significant challenge. This is particularly difficult in biomedicine, where POS tags serve as an input to training sophisticated literature summarization techniques, such as those based on Hidden Markov Models (HMM). Different approaches have been taken to deal with the POS tagger challenge, but with one exception – the TnT POS tagger - previous publications on POS tagging have omitted details of the suffix analysis used for handling unknown words. The suffix of an English word is a strong predictor of a POS tag for that word. As a pre-requisite for an accurate HMM POS tagger for biomedical publications, we present an efficient suffix prediction method for integration into a POS tagger.

**Results:**

We have implemented a fully functional HMM POS tagger using experimentally optimised suffix based prediction. Our simple suffix analysis method, significantly outperformed the probability interpolation based TnT method. We have also shown how important suffix analysis can be for probability estimation of a known word (in the training corpus) with an unseen POS tag; a common scenario with a small training corpus. We then integrated this simple method in our POS tagger and determined an optimised parameter set for both methods, which can help developers to optimise their current algorithm, based on our results. We also introduce the concept of counting methods in maximum likelihood estimation for the first time and show how counting methods can affect the prediction result. Finally, we describe how machine-learning techniques were applied to identify words, for which prediction of POS tags were always incorrect and propose a method to handle words of this type.

**Availability and Implementation:**

Java source code, binaries and setup instructions are freely available at http://genomes.sapac.edu.au/text_mining/pos_tagger.zip.

## Introduction

Hidden Markov Models (HMM) have been used in Part-Of-Speech (POS) tagging of text for 30 years. HMM and, more recently, Conditional Random Field (CRF) models [1] have been shown to be more accurate compared to other rule based methods such as [2], according to [3].

In the process of tagging articles, one always comes across new words. When training corpora are limited, this problem becomes more acute. Biology in particular, with its proliferation of new words and new gene ontology terms, requires a POS tagger with an efficient method to handle new words. The existence of special characters (capitals, numbers, hyphens or symbols) is the first characteristic used to predict a word tag. If a new word does not contain any special characters, particularly when that word is made of all alphabetic lower case characters, the best method to predict a word tag is to examine the lexical structure of the word, such as the suffix and postfix. In English and some other languages, the suffix is a strong predictive feature for word tagging. In this study we first implemented the TnT POS tagger as a standard machine learning tagger. We then used TnT’s suffix analysis method to handle new words. Subsequent testing of TnT system gave an unsatisfactory result for suffix analysis, prompting us to design and implement a novel method, which increased accuracy from 66 to 95 percent.

The problem of handling new words has previously been addressed by manually extending the lexicon by adding new words and all of their possible tags to existing lexicon, as in [4], and while this method seems to be simple and accurate, it requires ongoing effort to identify new biological words and add them to the lexicon. This is particularly problematic in the field of biology, where new chemical, biochemical and genetic terms are emerging in papers every day. So, for this study, we did not consider a lexicon-based method to be appropriate for POS tagging of new biological words. Instead, we focused on improving machine learning techniques for POS tagging, using word lexical features such as special characters and suffixes [5], [6]. We will show how we can achieve better performance by mixing this approach with our proposed machine leaning method.

### Hidden Markov Model Theory of POS Tagging

If a sentence of length N, contains words w_1_, w_2_…,w_N_, and POS tags for them are t_1_, t_2_…, t_N_, then according to the topology of the HMM the joint probability of this combination will be:
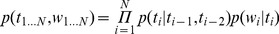
(1)


The first term is p(t_i_|t_i−1_,t_i−2_), and suggests that each word tag depends on 2 previous tags. This is known as a 3-gram HMM and has been chosen because it has been previously shown that 3-gramsare more accurate than 4-grams [7]. We can estimate this term by counting 3-gram frequencies, and for zero frequency 3-grams we use a previously described efficient smoothing algorithm [8].

The second term is p(w_i_ | t_i_), which determines the word probability distribution given a POS tag, and we refer to it from now on as the word conditional probability. This conditional probability shows that the probability of one observation (word) only depends on its current state (tag), not on previous or subsequent states.

To estimate this term, we needed first to process our training corpus. We calculated the frequency with which each word occurs in the corpus and built a *lexicon* database table to store those frequencies. For simplicity we show the *lexicon* database table with its fields defined below:
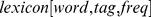
(2)


Subsequently, for each word in our lexicon we determined the maximum likelihood estimation (MLE):
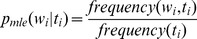
(3)Where the numerator is the number of times word w_i_ had tag t_i_ in our ontology database table and the denominator is the number of times that tag t_i_ was assigned to a word. Both of these were determined using *lexicon* database table.

Of course, our training corpus only contained a limited number of words, whereas our HMM system must be able to deal with text containing many words that do not exist in our *lexicon* database table (unseen words). Thus for unseen words, the P_mle_ will be zero and not applicable for equation 1.

In this situation, the most predictive features of a word’s tag in English and some other languages are its suffix and special characters. For example a word ending in ‘_ing’ can have tags VVG, VVJG and VVNG (see Table S1). In this paper we propose a solution to estimate the probability of a word with a particular suffix, having a particular tag. For example, we estimate p(Suffix = ing | Tag = VVG). In other words:
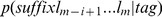
(4)


Then we propose a comprehensive character feature analysis and a method to interpolate suffix and character feature probabilities into a single probability to be used in equation (1). Wherever a word does not exist in our lexicon, P_mle_(word|tag) is zero. We have also used suffix analysis to determine the conditional probabilities of our lexicon words for unseen tags, and we show how efficient suffix information can be used to smooth word probabilities associated with all possible tags, particularly where the conditional probability of a word is based on sparse or unseen POS tags.

In this study, we first explain the previously published TnT method and describe its shortcomings, which led us to propose a simple method for estimating a word’s conditional probability. We have evaluated both approaches using real data, and can demonstrate that our method provides a significant improvement in accuracy. We also report optimal parameter settings for both methods.

We have also compared our POS tagger with another state-of-the-art POS tagger that is very well trained based on corpora from different fields including technical terms and these results confirm our POS tagger’s efficiency in tagging biological terms such as genes and protein names.

### Suffix Prediction Methods

#### 1 TnT/Probability interpolation [7]

The central concept in this method was first used in the original TnT POS-tagger, but some parameters are discussed in [7]. Here we explain these parameters and will show how to set them in our experiments.

If a word of length m ends with a suffix of length i, shown as l_m-i+1_…l_m_, then that word also ends in a suffix of length i−1, l_m-i+2_…l_m_, until the suffix length is 0.We therefore need to *interpolate* probabilities between suffix lengths i and i−1, to be able to derive the probability to be used in (4). First we estimated:
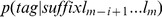
(5)


To estimate (5), we first determined the frequency of each suffix in our lexicon. In order to do this, we examined all words in *lexicon* database table, and counted the occurrences of each suffix. Finally we made a new database table for suffixes containing suffix, tag and frequency:
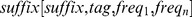
(6)


Here we used two counting methods and stored the results of both (*freq_1* and *freq_n*) in *suffix* database table. *freq_1* is the raw frequency, in that we do not multiply the frequency of the suffix by the frequency of the word itself in *lexicon* database table. For *freq_n,* we multiply the suffix frequency by the word frequency in *lexicon* database table. We know that for each suffix-tag pair:
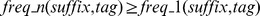



The counting method can affect the accuracy of different probability interpolation methods, and we demonstrate this below when applied to real datasets. In fact, our study is the first study to examine the effect of multiplier in counting for MLE estimations of suffix.

It is clear that we can re-state p_ml*e*_ in (5) as:
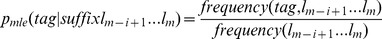
(7)


*p*_ml*e*_ can then be estimated using data from *suffix* database table. This probability is a proportion, because, p_mle_(tag = VVJ | suffix = ‘ing’) is equivalent to the proportion of words with suffix ‘ing’ that are tagged as VVJ, and we know that:
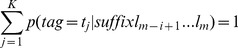
(8)
*Where k* is the maximum number of POS tags (in Table S1, *k* equals 60).

So for probability interpolation we can state:
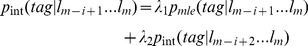
(9)Where



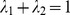



Equation 9 is recursive and starts from p(tag), meaning that the probability for a suffix with length 1 is interpolated with p(tag).As an initial condition, we assume:
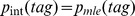



We continue interpolating until maximum length 5. [7] has proposed a maximum length of 10, but [4] have argued that well known English suffixes are not more than 4 characters in length and they have proposed using a maximum length of 4. Therefore we concluded that for English, 5 was a reasonable maximum length.

In the TnT/probability interpolation method, the estimation of coefficients λ_1_ and λ_2_ is based on the standard deviation of p_mle_(tag) for all tags, which usually yields values between 0.03 and 0.10.
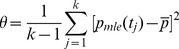
(10)
*Where k* is the number of POS tags, and in the example presented in Table S1 is 60.



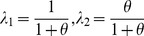
(11)In the TnT method, *θ* is calculated regardless of context, meaning that coefficients are fixed for all suffixes and tags. In addition, two parameters are not specified. The first parameter is the counting method, the values for which were stored in database table *suffix,* and referred to as *freq_1* and *freq_n*. The second parameter is the interpolation method, and is how suffixes are interpolated in 9. We propose 3 different interpolation methods, which reflect the degree or depth of interpolation. To illustrate these different methods we used a 2 dimensional array to store all of the MLE probabilities needed. For example in the word ‘tubulointerstitial’, we started from suffix ‘l’ up to suffix ‘itial’, we estimated the MLE probability and marked the array cells below if we had an entry for that suffix-tag pair in the ‘*suffix’* table:

For each column in Table 1 we can interpolate three ways:

**Table 1:** suffix versus tags for each suffix in *suffix* database table.

Suffix	POS tags											
	DB	VVI	II	CS	VM	NN	RR	NNP	PND	JJ	VVB	DD
**l**	freq(DB,l)	freq(VVI,l)	freq(CS,l)	freq(CS,l)	freq(VM,l)	freq(NN,l)	freq(RR,l)	freq(NNP,l)	freq(PND,l)	freq(JJ, l)	freq(VVB,l)	freq(DD,l)
**al**		freq(VVI,al)				freq(NN,al)	freq(RR,al)			freq(JJ,al)	freq(VVB,al)	
**ial**						freq(NN,ial)				freq(JJ,ial)		
**tial**						freq(NN,tial)				freq(JJ,tial)		
**itial**

1. We interpolate up to the maximum suffix length that has an entry in the *suffix* database table. In this example the suffix is ‘*tial*’ which has a length of 4, so depending on the entries in the Table 1, it could be any value from 1 to 5, in this example the maximum value is in column ‘JJ’.
2. We interpolate up to 5 levels regardless of the existence of a corresponding entry in the *suffix* database table.
3. We interpolate until we have an entry for that tag in the *suffix* database table. For example for column tag ‘VVI’, we interpolate for 2 levels.

After estimating the interpolated probability for each column of Table 1, we used the following Bayesian rule to estimate (4):
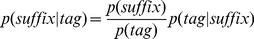
(12)


For *p(tag)*, we used the sum of the frequencies of all the rows in the *suffix* database table for that tag. For *p(suffix)*, we used the sum of frequencies of all the rows with a suffix of length 1 (the choice of length is arbitrary because it is the relative value for each tag that is important in equation 1). Suffixes of length 1 represent the maximum suffix frequency because suffix counts are cumulative. Therefore, in our example for the word ‘tubulointerstitial’, to calculate the probability ratio in (12), for a tag ‘NN’ we would use the following value:
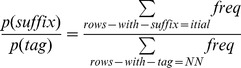
(13)


This allowed us to calculate the joint probability in (1) using the Viterbi algorithm [9].

#### 2 Maximum Suffix Length (MSL) method

This method differs from the suffix probability interpolation approach because it only requires the probability of the maximum length suffix for each tag. For this, we created a 2-dimensional array based on Table 1 as previous method. For example to estimate p(‘tubulointerstitial’| tag = JJ), we used Table 1, and for example it was apparent from column JJ that the best option was to use the suffix ‘*tial*’, which we were able to estimate using the *suffix* database table as shown below:
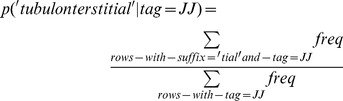
(14)


By estimating (14), we were able to use it directly in (2) by applying the Viterbi algorithm [9]. In the following sections we demonstrate the increased efficiency of MSL over the TnT probability interpolation method.

### Materials and Experimental Design

#### 1 Implementing POS tagger

We implemented a trigram HMM and used a linear interpolation method between unigram and bigram [7], [10]–[12] for smoothing. We trained our HMM using a tagged corpus available from NCBI [4], using maximum likelihood training for better performance [13].

We built our *lexicon* and *suffix* database tables based on frequencies acquired from the training corpus. Our database tables contained 20,662 *lexicon* word-tags (18,416 distinct words) and 16,004 *suffix* database table suffix-tag pairs. We used the set of POS tags defined in Table S1. We performed our suffix analyses to estimate the smoothed probability of all known words in our lexicon for unseen POS tags (as opposed to using the suffix analyser for unseen words), and updated *lexicon* database table for newly detected tags and their conditional probabilities.

We then analysed all the words in *lexicon* database table, for the occurrence of special characters (punctuation, Greek letters, digits, symbols, etc…) and lower/upper case letters. In order to do this, we assigned a feature string to each word, representing the type and order of characters used in that word. We then estimated the maximum likelihood probability for each feature string and stored that information in *char_ feature* database table, as we did for *suffix* database table*:*

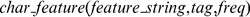
(15)


To estimate *P_mle_(word|tag)*, we first looked up each word in *lexicon* database table, for each match we used all the smoothed tag probabilities. If we couldn’t find a match, we retrieved the word’s character feature conditional probability *p_char_feature_* using *char_feature* database table. This probability was informative if the word contained upper case letters or numbers or other non-letter characters. But if the word was all lower case, it was not very helpful, so for this situation we used the suffix conditional probability *p_suffix_* from *suffix* database table. Once this process was complete, we had a value for *P_mle_(word|tag)*, that might have originated from *lexicon*, *suffix* or *char_feature* database tables, for use in equation (1). Finally, used the Viterbi algorithm [9] to tag the whole sentence.

#### 2 Experiment 1; suffix analysis comparison

In order to compare the MSL method to the probability interpolation method, we designed the following experiment. First, we applied the interpolation method with different values for two parameters: *Counting Method* and *Interpolation Method,* where *Counting Method* values 1 and 2 stand for *freq_1 and freq_n* and *Interpolation Method* has values 1, 2 and 3 as defined in section 2.1. This resulted in 2×3 different parameter combinations.

For the MSL method, only the *Counting Method* parameter changes the tagging result. We used *Counting Method* with values of 1 and 2, resulting in a total of 8 different parameter sets.

To prepare testing data, we downloaded biological articles from the NCBI website [14]. We randomly selected 450 articles from different biological journals, utilising the Viterbi algorithm [9] to tag sentences in these articles.

To determine which words should be tagged based on their suffixes we used the following method. First we checked words in a case independent fashion in the *lexicon* database table. If we found no match, we tested the word for known patterns, such as numbers, number ranges, ordered numbers, etc… We selected lowercase non-matching words that failed to contain known patterns for suffix analysis. We carried out the suffix analysis with our 8 different parameter sets for the two methods. We only recorded POS tag results that were discordant as a function of parameter or method, as we were interested in the relative performance of the two methods.

#### 3 Experiment 2; state-of-art stanford maxent tagger comparison

In the second experiment we compared the overall performance of our POS tagger, with a popular and mature POS tagger. We selected the Maxent POS tagger [5] for 2 reasons: first, this POS tagger has reported a higher accuracy in tagging unknown words and second, this POS tagger has been developed using Java, like our POS tagger. This POS tagger is based on a second order conditioning model and maximum entropy classifiers [6], and uses a cyclic dependency network. This POS tagger comes with different models and we selected the most complete and accurate model called ‘english-bidirectional-distsim’, which was trained based on Wall Street Journal (WSJ) data, extra English data and technical English data.

MaxentTagger uses The University of Pennsylvania (Penn) Treebank tag-set which consists of 45 POS tags, while our tag-set consists of 60 POS tags (see Table S1). These 2 tag sets have nearly the same POS categories but with different notations and our POS tags are more specific with respect to verbal forms. In order to make a fair comparison, we made a table that maps each tag from one tag-set to its equivalent in another tag-set. We then selected 20 randomly selected articles from NCBI (experiment 1), extracted sentences, and tagged them with both POS taggers.

The first difference we observed was related to the way the two taggers tokenized sentences. Our tokeniser was more accurate in detecting numbers, signs and complex biological names, particularly where a biological name contained special characters like a hyphen, parenthesis, slash, dot or other symbol. In many cases, MaxentTagger tended to split those words, and in some cases combined punctuation characters with the actual word, which led to incorrect results.

We excluded all the tokens that were tokenised differently by the two taggers and only compared the POS tags of similar words. We disregarded words with concordant tags and only logged words with discordant tags (using the mapping table connecting the two tag-sets), as we were interested in the relative performance of the two methods.

## Results

### 1 Experiment 1

After processing 450 biological articles, we tagged a total of 79,791 words based on suffix analysis, of those, 28,895 words with discordant POS tags were identified. We randomly selected a total of 1,500 words in 15×100 word samples, and manually corrected them, (Table S2) with a summary of the results shown in Table 2.

**Table 2:** Statistics in all 15 samples for each parameter set.

Main Method	CountingMethod	InterpolationMethod	Samples Mean(number of correct tags)	Totalcorrect tags	OverallAccuracy
MSL method	Freq_1	N.A.	88.46	1327	95.82%
MSL method	Freq_n	N.A.	88.86	1333	95.96%
Probability Interpolation Method	Freq_1	Method 1	3.4	51	65%
Probability Interpolation Method	Freq_1	Method 2	3.33	50	64.99%
Probability Interpolation Method	Freq_1	Method 3	7.2	108	66.39%
Probability Interpolation Method	Freq_n	Method 1	2.66	40	64.74%
Probability Interpolation Method	Freq_n	Method 2	2.4	36	64.65%
Probability Interpolation Method	Freq_n	Method 3	5.14	77	65.64%

We found that 88.86% of the discordant POS tags were correctly assigned using the maximum length method, compared to7.2% using the interpolation method. Overall (concordant plus discordant), the MSL method was nearly 50% (95.82% vs. 66.39%) more accurate than the interpolation method for suffix prediction. We have shown that the accuracy of suffix based POS tagging can be greater than 95.96% according to line 2 of Table 2.

### 2 Experiment 2

After processing 20 articles, we found 246 differentially tagged words where MaxentTagger was correct 48% of the time and our tagger was correct 52% of the time. MaxentTagger was better at detecting proper nouns (NNP) like city names, countries and persons, not surprising considering its comprehensive corpus, but our POS tagger was more accurate in tagging biological names. In many cases MaxentTagger incorrectly tagged biological names and symbols as FW (Foreign Word).

Considering the fact that our training corpus was significantly smaller and limited to biological texts, but that it still out performed MaxentTagger, we conclude our POS tagger was more efficient at tagging biological texts.

## Discussion

We have shown that the MSL method is much more accurate than the probability interpolation method for POS tagging biological words based on suffix analysis. The MSL method is relatively insensitive to the *Counting Method* parameter, but the *freq_n* multiplier method gave a slightly better result. We also have the optimum parameter selection for the interpolation method where *Counting Method* 1 (multiplier 1) and interpolation method 3, yielded the best result.

In addition to superior accuracy, the MSL method is much faster than the interpolation method, this is because it not only performs fewer calculations in equation (9), but also obviates the need for calculations required by equation 12. Because we stored our *lexicon* and *suffix* tables in a database, the MSL method required significantly less time for database access. Both methods exhibit linear time complexity, so they do not differ in that regard.

It should be mentioned that some words were incorrectly POS tagged in all 8 parameter sets. This shows that all machine learning methods failed to POS tag some unknown words. Surprisingly, these words are all common English words and none of them are specifically biological words. Fortunately they account for a very low percentage of all unknown words in biological articles (less than 1% in our experiment). These errors occurred because these common English words were similar to known suffixes in our lexicon. In Table 3, we have listed the problematic words we detected in our dataset.

**Table 3:** Words incorrectly tagged with all methods.

Word	Wrong Tag	Correct Tag
breathe	DD	VVI
comply	RR	VVI
kept	II	VVN
obese	DD	JJ
bring	VVG	VVI
kits	PNG	NNS

These unknown common English words are not actually unknown, but they were unknown to our lexicon, that was constructed based on our training corpus, so it makes sense to manually add them along with their POS tags to our lexicon as suggested in [4]. The work required to add new common English words is significantly less than for new biological words, since unknown common English words accounted only one percent of all of the unknown words encountered. For example in the case of irregular verbs (that don’t exist in the *lexicon* database table), of which there are about 190, we could simply add them to the *lexicon* database table. However, according to our results, predicting the POS tag of a new biological word is fairly accurate (more than 95.96% based on our results) using our machine learning method.

An alternative and more complicated machine learning method would be to use noun and verb phrases, based on grammar rules. For example, we could try to parse our sentences based on tags, allowing us to detect phrases that violate grammar rules, based on incorrect POS tags. We could then replace incorrect tags with more appropriate ones. This method is non-trivial and needs more research, but one possible approach might bet to use dynamic CRF [15] with tag and phrase information to train the CRF based on 2 features. We expect we could further reduce the number of POS tag errors significantly in this fashion.

Based on comparison of our POS tagger with MaxentTagger, we conclude that our tokenising method tokenised sentences much better than MaxentTagger’s tokeniser. Even though MaxentTagger was more accurate tagging common English words and proper nouns, our tagger was better at unknown biological names and gene ontology, due to combined MSL suffix and character feature analysis. Finally, we also showed the importance of suffix probabilities for smoothing the conditional probabilities of unseen POS tags based on known words from our lexicon.

## Supporting Information

Table S1 Table of POS tags used in our experiment.
(DOC)

Table S2 Table of 15 manually corrected word samples.
(DOC)
