# Supplementary material for: Improved Part-of-Speech Prediction in Suffix Analysis
Source: PLoS One. 2013 Oct 4;8(10):e76042. doi: 10.1371/journal.pone.0076042 (PMC3790802; doi:10.1371/journal.pone.0076042)
Supplement: Table S1 — Table of POS tags used in our experiment. (DOC) [file pone.0076042.s001.doc]

Table S1: Table of POS tags used in our experiment:

| CC coordinating conjunction | NNS plural noun | VBN participle been | VVD past tense |
| --- | --- | --- | --- |
| CS subordinating conjunction | PN pronoun | VBZ 3^rd^ present | VVG present part |
| CSN comparative conjunction | PND determiner as pronoun | VDB base do | VVI infinitive lexical verb |
| CST complementizer | PNG genitive pronoun | VDD past did | VVN past participle |
| DB predeterminer | PNR relative pronoun | VDG participle doing | VVZ 3^rd^ present |
| DD determiner | RR adverb | VDI infinitive do | VVNJ prenominal past part. |
| EX existential | RRR comparative adverb | VDN participle done | VVGJ pronominal present part. |
| GE genitive marker | RRT superlative adverb | VDZ 3^rd^ present | VVGN nominal gerund |
| II preposition | SYM symbol | VHB base have | ( left parenthesis |
| JJ adjective | TO infinitive marker | VHD past had | ) right parenthesis |
| JJR comparative adjective | VM modal | VHG participle having | , comma |
| JJT superlative adjective | VBB base be | VHI infinitive have | . end of sentence |
| MC number | VBD past was, were | VHN participle had | : colons |
| NN noun | VBG participle being | VHZ 3^rd^ present | `` left quote |
| NNP proper noun | VBI infinitive be | VVB base form lexical verb | ‘’ right quote |
