# Supplementary material for: Improved Part-of-Speech Prediction in Suffix Analysis
Source: PLoS One. 2013 Oct 4;8(10):e76042. doi: 10.1371/journal.pone.0076042 (PMC3790802; doi:10.1371/journal.pone.0076042)
Supplement: Table S2 — Table of 15 manually corrected word samples. (DOC) [file pone.0076042.s002.doc]

Table S2 :

| Sample | MSL, Freq_1 | MSL, Freq_n | PIM, Freq1,Int1 | PIM, Freq1, Int2 | PIM, Freq1, Int3 | PIM, Freq_n, Int1 | PIM, Freq_n, Int2 | PIM, Freq_n, Int3 |
| --- | --- | --- | --- | --- | --- | --- | --- | --- |
| 1 | 90 | 90 | 4 | 5 | 9 | 3 | 3 | 5 |
| 2 | 84 | 84 | 8 | 7 | 8 | 4 | 4 | 7 |
| 3 | 89 | 90 | 4 | 4 | 8 | 2 | 1 | 2 |
| 4 | 91 | 92 | 2 | 1 | 4 | 2 | 2 | 3 |
| 5 | 86 | 86 | 6 | 5 | 9 | 4 | 3 | 7 |
| 6 | 91 | 94 | 2 | 1 | 4 | 0 | 0 | 4 |
| 7 | 91 | 92 | 1 | 1 | 5 | 3 | 3 | 3 |
| 8 | 84 | 85 | 5 | 5 | 9 | 4 | 3 | 7 |
| 9 | 85 | 84 | 3 | 3 | 5 | 3 | 3 | 5 |
| 10 | 89 | 91 | 3 | 3 | 9 | 3 | 3 | 7 |
| 11 | 92 | 91 | 2 | 2 | 9 | 1 | 0 | 6 |
| 12 | 94 | 96 | 2 | 2 | 5 | 2 | 2 | 5 |
| 13 | 88 | 86 | 1 | 1 | 12 | 5 | 5 | 10 |
| 14 | 84 | 85 | 5 | 5 | 8 | 2 | 2 | 4 |
| 15 | 89 | 87 | 6 | 5 | 4 | 2 | 2 | 2 |

*MSL:* Maximum Suffix Length Method*PIM:*ProbabilityInterpolation Method
